# Exercise is the dominant factor affecting the development of teenagers' eyesight—Based on the Bayesian model averaging

**DOI:** 10.3389/fpubh.2022.1014227

**Published:** 2022-12-14

**Authors:** Zhong-hui Liu, Meng-fei Zhao, Shuai Ma, Yin Li, Zhi-ying Sun, Lei Gao

**Affiliations:** ^1^Tianjin Center for Disease Control and Prevention, Tianjin, China; ^2^Department of Maternal, Child & Adolescent Health, School of Public Health, Tianjin Medical University, Tianjin, China

**Keywords:** eyesight, Bayesian model averaging (BMA), influencing factors, weekly exercises, junior and senior middle school

## Abstract

**Objective:**

The model uncertainty may result in inconsistency about the environmental factors of myopia among students, and the Bayesian model average (BMA) is an effective way to eliminate it. We aimed to explore the influencing factors of myopia in primary and middle school students by BMA.

**Methods:**

The data came from the 2021 National Surveillance of Common Diseases and Health Influencing Factors of students. By stratified random cluster sampling, the physical and mental health status of students in Tianjin and the factors affecting their physical health, such as diet, exercise, mental stress, school bullying, sleep time, and internet use, were investigated. The sample consisted of 8,457 primary school students, 8,191 junior middle school students, and 5,901 senior middle school students. Besides the physical examination, we used computer optometry (non-ciliary paralysis) to screen myopia. And we used BMA to select the risk factors through the BMS package in R.

**Results:**

The exercise was the only factor that affected the eyesight of junior and senior middle schoolers by BMA, with the posterior probability of 0.9736 and 0.9762, but not for the primary students. And we failed to select variables that affected eyesight in grades 4–6 of primary school.

**Conclusion:**

The exercise was a strong influencing factor for the eyesight of students in Tianjin's junior and senior middle schools.

## Introduction

Refractive error is the significant cause of visual impairment and one of the key tasks of the global blindness prevention program (1, 2). According to the World Health Organization, refractive error is the most common cause of vision damage worldwide (3). Myopia is one of the most common refractive errors. In recent years, the prevalence of myopia has increased (4). Especially in Southeast Asia, some areas show that the prevalence rate of myopia in children is close to or even more than 50% (5–7). The rising incidence of myopia among students in Asia has become a major public health challenge (8). In China, the prevalence rate of myopia among junior middle school students increased from 55.95% in 2005 to 65.48% in 2015 (9). The prevalence of myopia among middle school students rose from 79.5% in 2001 to 87.7% in 2015 (10). The increasing prevalence of myopia in students affects their health, and increases the risk of eye complications (11, 12).

In recent years, many researchers have conducted a series of studies on the risk factors of myopia, especially the environmental factors, but the results remain inconsistent. For example, a cohort study in Singapore found that a higher intake of saturated fat and cholesterol was positively related to Axial length (13). However, another birth cohort study in Singapore showed that the total dietary fat intake at 6, 9, and 12 months was not related to myopia at the age of three (14). Another example is the role of outdoor activities. Some studies believe that outdoor activities are protective factors for eyesight. For example, a randomized clinical trial conducted in Guangzhou shows that increasing outdoor activities in schools could reduce the incidence of myopia in the next 3 years (15). However, another study in the same field found that outdoor activities were not the influencing factor of myopia (16). The inconsistency may result from methodological heterogeneity, such as participants, study design, and model uncertainty. As Raftery once pointed “the standard method of selecting a single model and reasoning based on it underestimates uncertainty of the quantities of interest because it ignores uncertainty of the model form” (17). The uncertainty of the model means that the unspecified model will lead to the deviation of the parameters, while the over-specified model will lead to a poor prediction effect of future samples. That is, there is a problem in the trade-off between dispersion and variance of the model (18). Undoubtedly, the uncertainty of the model will also affect the validation of myopia prevention factors. Therefore, scholars try to solve these problems through model combination or model averaging (19). The earlier developed method is Bayesian model averaging (BMA), which can judge whether it has a steady impact on the results by the posterior probability of parameters, and can screen and determine the key influencing factors of the results according to the posterior probability of different parameters (17). Many studies on ensemble prediction using the BMA method have shown that the prediction result of the BMA method is more reliable than that of a single model, and it has been widely used (20–23).

In addition, students of different learning periods may have varied burdens due to distinct academic requirements. Higher grades and school workload may contribute to the higher incidence of myopia. A study in East China found that the prevalence of myopia has been increasing from primary school to middle school, with the nonlinear increase in grades, the prevalence of myopia among senior middle school students has reached an alarming height, with 12% in kindergarten, 32% in grade two, 69% in grade five, and ~90% by grade 10 (the 1st year of senior middle school) or above (24). In addition, pubertal development probably plays a role in the progression of myopia. For example, one study once argued that puberty may play a role in the relationship between outdoor time and refractive development among Chinese children and adolescents (25). According to a study in Zhengzhou, most Chinese junior middle school students have entered puberty (26). Therefore, it is more reasonable to establish pupil-specific, junior-high-school-student-specific, and senior-high-school-student-specific prediction models to verify the influencing factors of myopia in each stage. Therefore, considering the uncertainty of the model and the influence of puberty on outdoor time, we aim to use the BMA method to select the influencing factors of Chinese students' refractive development in different school stages. In this way, we can take more targeted measures and provide a more scientific and accurate basis for preventing and controlling students' myopia.

## Methods

### Participants

**Figure 1:**
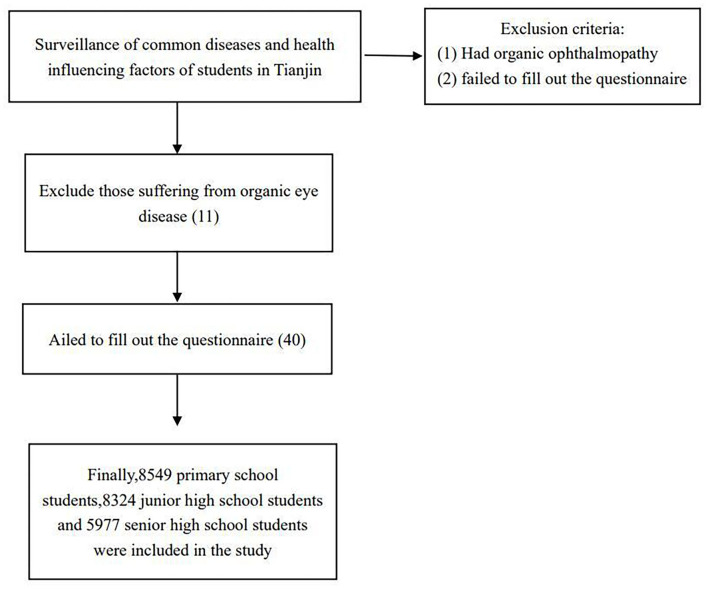
Flow chart of inclusion and exclusion.

Our data came from the National Surveillance of Common Diseases and Health Influencing Factors of Students in 2021 (Tianjin section). By stratified random cluster sampling, we investigated several common diseases (such as myopia, trachoma, conjunctivitis, dental caries, malnutrition, obesity, allergic asthma, iron deficiency anemia, parasitic diseases, mental disorders, abnormal curvature of the spine, etc.) and their health influencing factors every year. Our survey sample covered all administrative districts (seven urban and nine rural) in Tianjin, with two primary schools and two junior high schools in each administrative district (except high schools, two in each urban area but one in each rural area). We selected at least two classes (100 students) in each grade of every sample school (not including Grade 1 to Grade 3 in all primary schools). A total of 8,549 primary school students, 8,324 junior middle school students and 5,977 senior middle school students were included in the analysis. Students who had completed the vision test and questionnaire were eligible, and those who had organic ophthalmopathy and failed to fill out the questionnaire were excluded (see Figure 1). All the students involved in the survey signed the informed consent form.

### Myopia screening and refractive examination

Children's myopia screening mainly included refractive and hyperopia examination, and the criteria were the Guidelines for Prevention and Control of Myopia of Children and Adolescents (27). The visual acuity chart conformed to the national standard (GB 11533). According to this standard, we used the standard 5-meter logarithmic visual acuity chart to test the students' binocular vision, with the illuminance of 300 ~ 500 lx. The data on eyesight was based on the right eye vision. The refraction test used a desktop automatic computer optometry instrument that met the standard (ISO 10342 Ophthalmology Instrument -Optometry), and we used Topcon RM8000 (Japan) optometry in this survey. According to “Technical standard for physical examination for students (GB/T26343-2010)” and “Workbook of National Survey on Student Physical Fitness and Health in 2014”, students' eyesight was recorded by the numerical value of standard logarithmic eyesight. Also, the values of “spherical lens”, “cylindrical lens” and “axial position” in the automatic optometry results were also recorded. In the current study, we used the logarithmic vision as the outcome variable. All data preprocessing and analyses were conducted in R (version 3.6.2; R Development Core Team) within the RStudio (version 1.1.456) platform.

### Influencing factors

We mainly obtained the risk factors of myopia from the recent literature (28–31), such as the intake of nutrition and food supplements, which are often related to the progression of myopia. Also, daily activities, such as the time spent exercising, sleeping and surfing the internet with electronic devices, may affect children's eyesight. In addition, mental stress was once considered as the consequence and cause of vision loss (32). Meanwhile, we also investigated mental stress and school bullying, which were usually accompanied by mental stress (33). See the following:

1. Dietary factors: How many sugary drinks, fried foods, sweets, fresh fruits, and vegetables you had eaten in the past seven days, whether you have breakfast every day, etc.
2. Weekly exercise: How many days in the past week did you have at least 60 min of moderate-intensity activities, such as running, swimming, basketball, football, and so on?
3. Duration of Internet use: mainly included the average daily online time in the past week, the main content of surfing the Internet, the impact of surfing the Internet on study and life, and the desire to surf the Internet.
4. School bullying: what degree of bullying had students experienced in or around the school in the past 30 days?
5. Mental stress: center for Epidemiologic Studies Depression Scale (CES-D) (34) was adopted to evaluate the mental stress of participants (except the pupils).
6. Sleep duration: how many hours did students sleep every night on average?

### Investigation

With the assistance of the class teacher, we delivered the informed consent to the students and their parents in the early stage. All the investigators are trained in a unified way, taking the class as a unit, and answering questions anonymously in a collective and unified way. Participants completed it independently, and all questionnaires were collected on-site.

### Model settings and methods

In a linear model structure:


(1) $$\begin{array}{l} y=\alpha_{\gamma }+X_{\gamma }\beta_{\gamma }+\varepsilon \;\varepsilon \sim N\left(0,\sigma^{2}I\right) \end{array}$$

Among them, “y” represents the visual acuity of the right eye of school students, “β_γ_” is the coefficient vector to be estimated, “X_γ_” represents the factor affecting the visual acuity, “α_γ_” is a constant, and “ε” represents the independent and identically distributed random error term.

Suppose “y” is a quantity we are interested in, and data D is our survey data such as: diet score, exercise status. Let *M*= *{M*_1_*, M*_2_*, …M*_*K*_*}* represent a series of considered models, when there are “s” independent variables, which means that there are “K = 2^s^” models in total, the posterior distribution of “y” is obtained according to the Bayesian model averaging theory as


(2) $$\begin{array}{l} p\left(y|D\right)=\sum_{k=1}^{k}P\left(M_{k}|D\right)P\left(y|M_{k},D\right) \end{array}$$

where *P*(*y|M*_*k*_*,D*) is the posterior distribution of “y” given in the model “*M*_*K*_”, and *P*(*M*_*k*_*|D*) is the probability that “*M*_*K*_” is the optimal model. That is, the posterior distribution of “y” is actually the posterior probability as the weight, and the value obtained by weighting the posterior distribution of all models. In equation (2), assuming that “*M*_*K*_” is the optimal model, the probability distribution is:


(3) $$\begin{array}{l} P\left(y|M_{K,}D\right)=\int P\left(y|\beta ,M_{k},Z\right)P\left(\beta |M_{K,}D\right)d\beta \end{array}$$

β = (β_0_,β_1_,β_2_…β_n_), is the regression coefficient vector of the model “*M*_*K*_”, the model posterior probability *P*(*M*_*k*_*|D*) generated by Bayes' theorem is


(4) $$\begin{array}{l} P\left(M_{k}|y,X\right)=\frac{P\left(y|M_{k,}X\right)P\left(M_{K}\right)}{P\left(y|X\right)}\frac{P\left(y|M_{k},X\right)P\left(M_{k}\right)}{\sum_{s=1}^{k}P\left(y|M_{s}X\right)P\left(M_{s}\right)} \end{array}$$

Among them, *P*(*M*_*k*_) is the prior probability that the model “*M*_*K*_” is the optimal model (generally average, no information prior); *P*(*y*|*M*_*k*_, *X*) is the marginal likelihood value of the model “*M*_*K*_”, which is defined as


(5) $$\begin{array}{l} P\left(y|M_{k,}X\right)=\int P\left(y|\beta ,M_{k,}x\right)P\left(\beta |M_{k,}X\right)d\beta \end{array}$$

Among them, *P*(*y*|β, *M*_*k*_, *x*) is the likelihood function corresponding to the model “*M*_*K*_”, and *P*(β|*M*_*k*_, *X*) is the prior probability distribution of the parameter vector “β” in the model “*M*_*K*_”. Taking the posterior probability of the model as a weight, the posterior probability, mean and variance of the parameter vector “β_s_” can be calculated by using the weighted average


(6) $$\begin{array}{l} P\left(\beta |y,X\right)=\sum_{s=1}^{k}P\left(\beta |M_{s},y,X\right)P\left(M_{s}|y,X\right) \end{array}$$

(7) $$\begin{array}{l} E\left(\beta |y,X\right)=\sum_{s=1}^{k}E\left(\beta |M_{s},y,X\right)P\left(M_{s}|y,X\right) \end{array}$$

(8) $$\begin{array}{l} Var\left(\beta |y,X\right)=\sum_{s=1}^{k}Var\left(\beta |M_{s,}y,X\right)P\left(M_{s}|y,X\right) \end{array}$$

According to the posterior probability inference rule of BMA (35, 36):

P(β| y,X) < 0.5 means that there is no evidence to prove that the variable x is an influencing factor of visual acuity; 0.5 < P(β| y, X) < 0.75 means that there is weak evidence to prove that variable “x” is an influencing factor of visual acuity; 0.75 < P(β| y,X) < 0.95 indicates that there is strong evidence to prove that variable “x” is an influencing factor of vision; P(β| y, X) ≥ 0.95 indicates that there is strong evidence that the variable “x” is an influencing factor of visual acuity.

## Results

### Demographics

The final sample consisted of 8,457 primary school students, 8,191 junior middle school students, and 5,901 senior middle school students. As far as elementary school was concerned, we only included the children in grades 4–6, with an average age of 10.70 ± 0.91 years old. However, as to junior middle school and senior middle school, we covered all three grades with an average age of 13.69 ± 0.91 and 16.65 ± 0.96 years. As far as the gender composition was concerned, there were 4,424 boys and 4,033 girls in the primary school, 4,306 boys and 3,885 girls in the junior middle school sample, and 3,073 boys and 2,828 girls in the senior middle school part. See Tables 1, 2 showed the average values of relevant variables in each segment.

**Table 1:** The demographic and visual acuity of the final sample.

**Stages**	**Gender (*N*)**	**Age (mean ±SD)**	**Mean_Eyesight_**	**SD**
**Primary (grade 4–6 only)**				
Male	4,424	10.70 ± 0.91	4.82	0.56
Female	4,033		4.76	0.57
**Junior middle school**				
Male	4,306	13.69 ± 0.91	4.65	0.61
Female	3,885		4.54	0.61
**Senior middle school**				
Male	3,073	16.65 ± 0.96	4.51	0.62
Female	2,828		4.41	0.61

SD refers to standard deviation.

**Table 2:** Average value of relevant variables.

**Stage**	**Variable**	**Mean**	**SD**
Primary			
	Exercise	2.88	1.02
	Diet	9.13	1.41
	Bullying	10.78	1.44
	Sleep	8.86	1.10
	Network	9.00	1.82
Junior			
	Exercise	2.72	1.01
	Bullying	10.66	1.35
	M-stress	30.11	8.68
	Network	17.43	1.10
	Diet	9.43	1.43
Senior			
	Exercise	2.31	1.039
	Bullying	10.46	1.08
	M-stress	31.34	8.95
	Network	17.2	1.39
	Diet	9.67	1.37

Primary, primary school (grade 4–6); junior, junior middle school; senior, senior middle school; network refers to Duration of Internet use.

### Estimation results

#### Posterior model distribution

**Figure 2:**
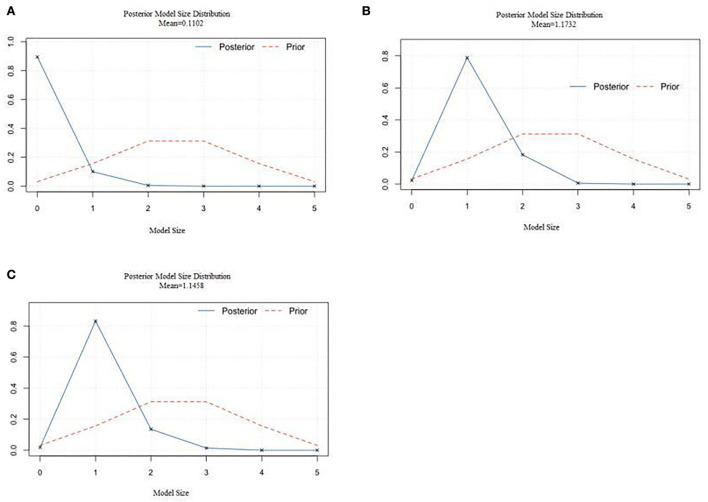
The mean value of the regular distribution of the posterior model for students. **(A)** The posterior model size distribution for primary school students. **(B)** The posterior model size distribution for junior high school students. **(C)** The posterior model size distribution for senior high school students.

Figure 2A showed the mean distribution of the posterior model for grades 4–6 in elementary school. The posterior probability in most models was < 0.75, and no variables were included. Figure 2B showed the mean distribution of the regular distribution of the posterior model in junior middle schools. The prior probability distribution was uniform, and the posterior probability of the model achieved a maximum value at the abscissa of 1, which meant the model only contains one explanatory variable. Similarly, Figure 2C showed the mean distribution of the regular distribution of the posterior model in senior middle school. The prior probability distribution was uniform, and the posterior probability of the model reached a maximum value at the abscissa of 1, again, weekly exercise was the only explanatory variable.

#### Estimation results of explanatory variables

Table 3 showed the BMA estimation results of explanatory variables for each grade. The posterior probability represents the explanatory power of the influencing factors. PIP represents posterior inclusion probabilities–i.e., the sum of PMPs for all models wherein a covariate was included. The agreement of the posterior mean represents the direction of action of the influencing factors. Post Mean displays the coefficients averaged over all models. The coincidence rate indicates that among all the model regression results of an explanatory variable, the coefficient of this variable is consistent with the same possibility as the posterior mean, which means the robustness of the variable. In Table 3, we can see that the five variables included in primary school, include physical activity in 1 week, diet score, injury score, sleep duration, and eye use in school. In the included model, the posterior probability values are all < 0.1, which means that these variables have no high explanatory power relative to visual acuity results, and there is no statistically significant. It indicated that these variables are not the main factors affecting primary school students' vision. For junior and senior middle school students, only weekly exercise was significant among the five variables including weekly exercise, diet score, injury score, psychological score, and Internet usage. The posterior probability of weekly exercise for junior middle school students was 0.9736, and the post mean is 0.017, which indicated that exercise can greatly prevent the further development of vision.

**Table 3:** BMA estimation results of explanatory variables for each grade.

**Stage**	**Variable**	**PIP**	**Post mean**	**Post SD**	**Sig**.
Primary					
	Exercise	0.04314520	0.00025010	0.00137775	1.00000000
	Diet	0.03013392	0.00010860	0.00075287	1.00000000
	Bullying	0.01374658	0.00002326	0.00034513	1.00000000
	Sleep	0.01229521	0.00002265	0.00044519	1.00000000
	Network	0.01083281	0.00000191	0.00020133	0.02775695
Junior					
	Exercise	0.97367226	0.01767462	0.00533409	1.00000000
	Bullying	0.15873106	0.00125250	0.00318150	1.00000000
	M-stress	0.01494183	0.00000462	0.00007739	0.86973570
	Network	0.01349785	0.00002033	0.00048775	0.14526280
	Diet	0.01234092	0.00000480	0.00034483	0.81741870
Senior					
	Exercise	0.97622593	0.01961372	0.00137775	1.00000000
	Bullying	0.08615652	0.00081861	0.00075287	1.00000000
	M-stress	0.05551302	−0.0000572	0.00034513	1.00000000
	Network	0.01430832	−0.0000147	0.00044519	1.00000000
	Diet	0.01357077	0.00000090	0.00020133	0.02775695

Primary, primary school (grade 4–6); Junior, Junior middle school; Senior, senior middle school; network refers to Duration of Internet use.

For senior high school students, we came to the same conclusion. The posterior probability of weekly exercise was 0.9762, and the post mean was 0.019, which also meant that exercise can prevent the further development of vision for senior middle school students.

**Figure 3:**
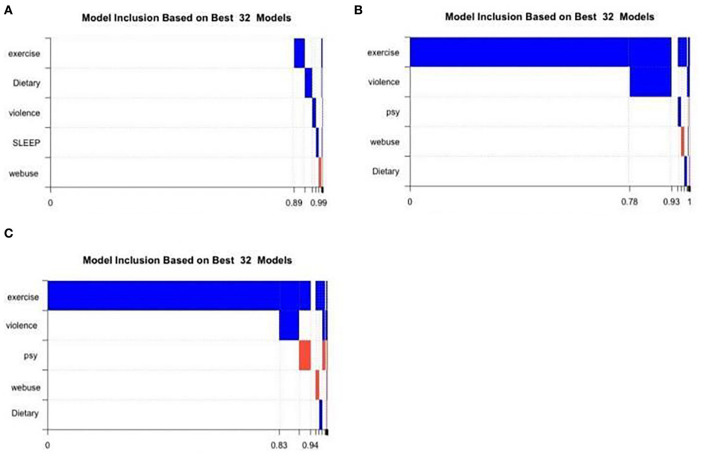
Variables inclusion in the 32 models of different stages. Violence refers to “school bullying”; psy refers to “mental stress”. **(A)** Cumulative model probabilities of primary school students. **(B)** Cumulative model probabilities of junior high school students. **(C)** Cumulative model probabilities of high school students.

For primary school students, weekly exercise was not included. None of the five variables was chosen in the best model (model 1), with a post-probability of 0.8947 (see Table 4). The best model for junior high school students only contains one variable (weekly exercise), and the posterior probability of the model was 0.7847. When we tried to include the second factor (injury), the posterior probability of the model became 0.1496. Similarly, among senior middle school students, the best model still included only one variable (weekly exercise). Figures 3A–C also showed the variables included in the rankings of the model in each stage. Blue represents a positive correlation, while orange represents a negative correlation. In addition, weekly exercise is the most included variable among the 32 junior and senior middle school models, while the five variables in the primary school model were not selected.

**Table 4:** Model post probabilities for each grade.

**Grade**	**Variable**	**Model 1**	**Model 2**	**Model 3**
Primary				
	Diet	0.00000000	0.00000000	1.00000000
	Exercise	0.00000000	1.00000000	0.00000000
	School bullying	0.00000000	0.00000000	0.00000000
	Network	0.00000000	0.00000000	0.00000000
	Sleep	0.00000000	0.00000000	0.00000000
	PMP (exact)	0.8947561	0.03974616	0.02725783
	PMP (MCMC)	0.8947561	0.03974616	0.02725783
Junior				
	M-stress	0.00000000	0.00000000	0.00000000
	Network	0.00000000	0.00000000	0.00000000
	School bullying	0.00000000	1.00000000	0.00000000
	Exercise	1.00000000	1.00000000	0.00000000
	Diet	0.00000000	0.00000000	0.00000000
	PMP (exact)	0.7847708	0.1496787	0.02202285
	PMP (MCMC)	0.7847708	0.1496787	0.02202285
Senior				
	Diet	0.00000000	0.00000000	0.00000000
	Exercise	1.00000000	1.00000000	1.00000000
	School bullying	0.00000000	1.00000000	0.00000000
	Network	0.00000000	0.00000000	0.00000000
	M-stress	0.00000000	0.00000000	1.00000000
	PMP (exact)	0.8272767	0.07081034	0.04064122
	PMP (MCMC)	0.8272767	0.07081034	0.04064122

Primary, primary school (grade 4–6); Junior, Junior middle school; Senior, senior middle school; network refers to Duration of Internet use.

## Discussion

On the basis of considering the model uncertainty, we used the Bayesian model averaging method to analyze the factors that affect the eyesight of primary school students, junior middle school students and senior middle school students. We found that exercise is the robust influencing factor that affects the eyesight of (junior and senior) middle school students, which has been consistent with previous studies (4, 37–40). However, few studies have identified the school stages (primary, junior or senior middle school) in the association between physical activities and myopia. Our results showed that in the progression of myopia, it is middle school students, not primary school students, who were mainly influenced by exercise. And this has deepened our understanding of the role of exercise in preventing myopia in distinct school stages. Our research results indirectly confirmed the prevention and control strategy of “Sunshine exercises for One Hour” for juvenile myopia in China (41), but it brought a new question: Are the factors that affect the progression of eyesight equally for students at different developmental stages and academic burdens?

Usually, the academic burden of junior high school students is much heavier than that of primary school students, which means that the duration of using eyes is longer and the exercise time is less. Meanwhile, middle-school students, compared to elementary-school students, show significant changes in almost all dimensions (academic, social, family, and physical) of self-conception (42), which leads to tremendous psychological and behavioral changes in individuals. And these changes possibly affect the effect of exercises on myopia. In addition, puberty also plays a role in the refractive development and its interaction with outdoor time, for example, one study once reported that puberty may play a regulating role in the relationship between outdoor time and refractive development among Chinese children and adolescents (25). Thus, we should consider the influential factors of intervention for juvenile myopia, such as academic burden, physical growth, psychological changes, and the external environment. It is necessary to conduct more trials aimed at the effect and possible mechanism of exercises on myopia by adopting a randomized controlled intervention experimental design in primary and middle schools.

Meanwhile, some critical questions remained unsolved about the correlation between exercise and myopia: Is there any distinction between physical activity and outdoor exposure? One systematic review showed that outdoor time was the most significant factor for myopia, but physical activity was not an independent risk factor for myopia (4). Similarly, an overview of systematic reviews and meta-analyses also reported that physical activity presented no effect on myopia, but increased outdoor time was protective against myopia development among non-myopes (40). Therefore, we inferred that physical activity and outdoor exposure may represent different mechanisms. However, Lingham et al. once reviewed the evidence of outdoor time on myopia and concluded that physical activity was one facet of spending time outdoors that may protect against myopia (38). The inconsistency of physical activity on myopia possibly originated from the lack of standard and objective measurements of physical activity apart from high heterogeneity of the type of studies, population sizes, ethnicity, or the age of study subjects. In the current study, we defined “exercise” as “at least 60 min of moderate-intensity activities, such as running, swimming, basketball and so on”, and we further quantified the “exercise” by how many days in 1 week. We failed to define “weekly exercise” as outdoor activity explicitly, but it is easy to deduce that, due to our specific purpose of investigating the efficacy of “Sunshine exercise for 1 h” in China and the fact that most sports listed in our definition are outdoor sports. Our definition of “exercise” seemed more accurate than before in terms of duration (at least 60 min), intensity (moderate-intensity), and frequency (how many days in 1 week), which made our results more instructive on the prevention and control of myopia among students.

Our results emphasize that middle school students should exercise for more than 60 min every week on the days of moderate and high intensity. The possible mechanism of a 1 h sunshine exercise to prevent myopia included that outdoor sunshine during exercise could promote dopamine secretion, slow down eyeball elongation and reduce myopia rate (43), which have been confirmed by animal experiments, and the protective effects of bright light on myopia development has been replicated in laboratory conditions (44).

Interestingly, our results only included exercise as the influencing factor of the vision among junior and senior middle school students, that did not mean that the rest variables had no effect. In fact, BMA model is regarded as superior at the variable selection (45, 46). For example, Wang et al. once compared the BMA approach with the stepwise procedures for selection of predictor variables in logistic regression using simulated data sets and the Framingham Heart Study data, and found that in most cases BMA selected the correct model and out-performed stepwise approaches (47). Therefore, our results can only prove that in primary school, the role of Sunshine exercise for 1 h is not more prominent than other factors; or some other important factors have been ignored. So, we need to further investigate the risk factors of myopia among primary school students, and make further analysis after including the students in grades 1–3, so as to find out the dominant influencing factors at this stage. However, in junior and senior high school, the role of exercise became more robust than other factors. Our findings provided a basis for accurate prevention and treatment of myopia among junior and senior high school students, and are expected to help improve their eyesight. To our best knowledge, BMA has not been introduced into the field of prevention and treatment of myopia in Chinese children yet. We strongly suggest that BMA should be used as a formal method for precise prevention and control of myopia.

There were several limitations in the current study. Firstly, we didn't include the students of grades 1–3 in the sample of primary school students, which may affect the research on myopia risk factors of primary school students. Secondly, the conclusion is limited among primary school students because paralytic mydriasis is not used. We will collect the data after mydriasis in future work, and then use the BMA model to select the influencing factors. Therefore, we should be careful when drawing conclusions among primary school students. Thirdly, we did not focus on a certain type of exercise that affected myopia progression, because we only wanted to learn the effect of “sunshine exercise for 1 h”, not a specific exercise. Finally, our definition of exercise intensity was self-rated and lacked objective data. In future research, we can subdivide the specific exercise type and availability of sports places, with some physiological data (such as blood pressure, heart rate, or pulse), to further explore the protective effect of exercise against myopia.

## Conclusion

Our results showed that “Sunshine exercises for 1 h” was the dominant factor provide a more scientific that affected the eyesight of students in junior and senior middle school in Tianjin, China. As far as the pupils are concerned, “Sunshine exercises for 1 h” has not shown any advantages over other interventions, at least among primary school students in grades 4–6.

## Data availability statement

The data that support the findings of this study are available from Tianjin Center for Disease Control and Prevention but restrictions apply to the availability of these data, which were used under license for the current study, and so are not publicly available. Requests to access the datasets should be directed to Z-hL, liuzhonghui@tj.gov.cn.

## Ethics statement

The studies involving human participants were reviewed and approved by Ethics Committee of Tianjin Center for Disease Control and Prevention. Written informed consent to participate in this study was provided by the participants' legal guardian/next of kin.

## Author contributions

Z-hL and Z-yS investigated and collected data. M-fZ wrote the manuscript. SM and YL consulted relevant literature. LG statistically processed the data and revised the article. All authors contributed to the article and approved the submitted version.

## References

[1] World Health Organization. (2019). Available online at: https://www.who.int/news/item/08-10-2019-who-launches-first-world-report-on-vision (accessed October 9, 2019).

[2] Rayapoullé A Gronfier C Forhan A Heude B Charles MA Plancoulaine S. Longitudinal association between sleep features and refractive errors in preschoolers from the EDEN birth-cohort. Sci Rep. (2021) 11:9044. 10.1038/s41598-021-88756-w 33907290 PMC8079679

[3] Ward DS Evenson KR Vaughn A Rodgers AB Troiano RP. Accelerometer use in physical activity: best practices and research recommendations. Med Sci Sports Exerc. (2005) 37:S582–8. 10.1249/01.mss.0000185292.71933.91 16294121

[4] Suhr Thykjaer A Lundberg K Grauslund J. Physical activity in relation to development and progression of myopia - a systematic review. Acta Ophthalmol. (2017) 95:651–9. 10.1111/aos.13316 27966836

[5] Rudnicka AR Kapetanakis VV Wathern AK Logan NS Gilmartin B Whincup PH. Global variations and time trends in the prevalence of childhood myopia, a systematic review and quantitative meta-analysis: Implications for aetiology and early prevention. Br J Ophthalmol. (2016) 100:882–90. 10.1136/bjophthalmol-2015-307724 26802174 PMC4941141

[6] Hsiao M Malhotra A Thakur JS Sheth JK Nathens AB Dhingra N. et al. Road traffic injury mortality and its mechanisms in India:Nationally representative mortality survey of 11 million homes. BMJ Open. (2013) 3:e002621. 10.1136/bmjopen-2013-002621 23959748 PMC3753525

[7] Williams KM Bertelsen G Cumberland P Wolfram C Verhoeven VJ Anastasopoulos E. Increasing prevalence of myopia in Europe and the impact of education. Ophthalmology. (2015) 122:1489–97. 10.1016/j.ophtha.2015.03.018 25983215 PMC4504030

[8] Wu PC Huang HM Yu HJ Fang PC Chen CT. Epidemiology of myopia. The Asia-Pacific J Ophthalmol. (2016) 5:386–93. 10.1097/APO.0000000000000236 27898441

[9] Li Y Liu J Qi P. The increasing prevalence of myopia in junior middle school students in the Haidian District of Beijing, China: a 10-year population-based survey. BMC Ophthalmol. (2017) 17:88. 10.1186/s12886-017-0483-6 28606071 PMC5468969

[10] Chen M Wu A Zhang L Wang W Chen X Yu X. The increasing prevalence of myopia and high myopia among middle school students in Fenghua city, eastern China: a 15-year population-based survey. BMC Ophthalmol. (2018) 18:159. 10.1186/s12886-018-0829-8 29970057 PMC6029024

[11] Recko M Stahl ED. Childhood myopia: epidemiology, risk factors, and prevention. Missouri Med. (2015) 112:116–21. 25958656 PMC6170055

[12] Flitcroft DI. The complex interactions of retinal, optical and environmental factors in myopia aetiology. Prog Retin Eye Res. (2012) 31:622–60. 10.1016/j.preteyeres.2012.06.004 22772022

[13] Lim LS Gazzard G Low YL Choo R Tan DT Tong L. Dietary factors, myopia, and axial dimensions in children. Ophthalmology. (2010) 117:993–997. 10.1016/j.ophtha.2009.10.003 20079928

[14] Chua SY Sabanayagam C Tan CS Lim LS Toh JY Chong YS. Diet and risk of myopia in three-year-old Singapore children: the GUSTO cohort. Clin Exp Optom. (2018) 101:692–9. 10.1111/cxo.12677 29577442

[15] He M Xiang F Zeng Y Mai J Chen Q Zhang J. Effect of time spent outdoors at school on the development of myopia among children in China: a randomized clinical trial. JAMA. (2015) 314:1142–1148. 10.1001/jama.2015.10803 26372583

[16] Guo L Yang J Mai J Du X Guo Y Li P. Prevalence and associated factors of myopia among primary and middle school-aged students: a school-based study in Guangzhou. Eye (Lond). (2016) 30:796–804. 10.1038/eye.2016.39 26965016 PMC4906452

[17] Raftery Adrian E. Bayesian model selection in social research. Sociol Methodol. 25:111–163. 10.2307/271063

[18] Kaplan D. On the quantification of model uncertainty: a bayesian perspective. Psychometrika. (2021) 86:215–38. 10.1007/s11336-021-09754-5 33721184 PMC7958145

[19] Claeskens G Hjort NL. Model Selection and Model Averaging || Frequentist and Bayesian Model Averaging. Cambridge: Cambridge University Press (2008) p. 192–226

[20] Pan H Yuan Y A. default method to specify skeletons for Bayesian model averaging continual reassessment method for phase I clinical trials. Stat Med. (2017) 36:266–79. 10.1002/sim.6941 26991076 PMC5026535

[21] Zhu G Li X Zhang K Ding Z Han T Man J. Multi-model ensemble prediction of terrestrial evapotranspiration across north China using Bayesian model averaging. Hydrol Process. (2016) 30:2861–79. 10.1002/hyp.10832

[22] Olson R Fan Y Evans JP. A simple method for Bayesian model averaging of regional climate model projections: application to southeast Australian temperatures. Geophys Res Lett. (2016) 43:7661–9. 10.1002/2016GL069704

[23] Zeng X Wu J Wang D Zhu X Long Y. Assessing Bayesian model averaging uncertainty of groundwater modeling based on information entropy method. J Hydrol. (2016) 538:689–704. 10.1016/j.jhydrol.2016.04.038

[24] Wang J Ying GS Fu X Zhang R Meng J Gu F. Prevalence of myopia and vision impairment in school students in Eastern China. BMC Ophthalmol. (2020) 20:2. 10.1186/s12886-019-1281-0 31898504 PMC6941318

[25] Wang J Cheng T Zhang B Xiong S Zhao H Li Q. Puberty could regulate the effects of outdoor time on refractive development in Chinese children and adolescents. Br J Ophthalmol. (2021) 105:191–7. 10.1136/bjophthalmol-2019-315636 32299828 PMC7848068

[26] Xing-cun Yao. Tracing Examine of Associations Between Early Puberty Timing and Obesity in Adolescent (master's thesis). Zhengzhou (IL): Zhengzhou University, MA thesis. (2018).

[27] National Health Commission of the PRC. (2019). Available online at: http://ww.nhc.gov.cn/jkj/s5898bm/201910/c475e0bd2de444379402f157523f03fe.shtml (accessed October 15, 2019).

[28] Fan Q Wang H Kong W Zhang W Li Z Wang Y. Online learning-related visual function impairment during and after the COVID-19 pandemic. Front Public Health. (2021) 9:645971. 10.3389/fpubh.2021.645971 34912766 PMC8666689

[29] Morgan IG French AN Ashby RS Guo X Ding X He M. The epidemics of myopia: aetiology and prevention. Prog Retin Eye Res. (2018) 62:134–49. 10.1016/j.preteyeres.2017.09.004 28951126

[30] Schuster AK Krause L Kuchenbäcker C Prütz F Elflein HM Pfeiffer N. Prevalence and time trends in myopia among children and adolescents. Dtsch Arztebl Int. (2020) 117:855–60. 10.3238/arztebl.2020.0855 33612155 PMC8025934

[31] Xiang ZY Zou HD. Recent epidemiology study data of myopia. J Ophthalmol. (2020) 2020:4395278. 10.1155/2020/4395278 33489329 PMC7803099

[32] Sabel BA Wang J Cárdenas-Morales L Faiq M Heim C. Mental stress as consequence and cause of vision loss: the dawn of psychosomatic ophthalmology for preventive and personalized medicine. EPMA J. (2018) 9:133–60. 10.1007/s13167-018-0136-8 29896314 PMC5972137

[33] Juvonen J Graham S. Bullying in schools: the power of bullies and the plight of victims. Annu Rev Psychol. (2014) 65:159–85. 10.1146/annurev-psych-010213-115030 23937767

[34] Radloff LS. The CES-D scale: a self-report depression scale for research in the general population. Appl Psychol Meas. (1977) 1:385–401. 10.1177/01466216770010030623302475

[35] Kass RE Raftery AE.Bayes factors. J Am Stat Assoc. (1995) 90:773-795.

[36] Zhang ZJ Peng WX Zhou YB Zhuang JL Jiang QW. The basic principle of Bayesian model average and its application in logistic regression. Chin J Health Statist. (2007) 32:467–71. 10.3969/j.issn.1002-3674.2007.05.006

[37] Bremond-Gignac D. Myopie de l'enfant [Myopia in children]. Med Sci (Paris). (2020) 36:763–8. 10.1051/medsci/2020131 32821053

[38] Lingham G Mackey DA Lucas R Yazar S. How does spending time outdoors protect against myopia? A review. Br J Ophthalmol. (2020) 104:593–9. 10.1136/bjophthalmol-2019-314675 31722876

[39] Zhu Z Chen Y Tan Z Xiong R McGuinness MB Müller A. Interventions recommended for myopia prevention and control among children and adolescents in China: a systematic review. Br J Ophthalmol. (2021). 10.1136/bjophthalmol-2021-319306. [Epub ahead of print]. 34844916

[40] Karthikeyan SK Ashwini DL Priyanka M Nayak A Biswas S. Physical activity, time spent outdoors, and near work in relation to myopia prevalence, incidence, and progression: an overview of systematic reviews and meta-analyses. Indian J Ophthalmol. (2022) 70:728–39. 10.4103/ijo.IJO_1564_21 35225506 PMC9114537

[41] Ministry of Education of China. (2020). Available online at: http://wap.moe.gov.cn/s78/A17/s7059/201410/t20141021_178927.html (accessed October 3, 2020).

[42] Onetti W Fernández-García JC Castillo-Rodríguez A. Transition to middle school: Self-concept changes. PLoS ONE. (2019) 14:e0212640. 10.1371/journal.pone.0212640 30785933 PMC6382118

[43] Cui D Trier K Munk Ribel-Madsen S. Effect of day length on eye growth, myopia progression, and change of corneal power in myopic children. Ophthalmology. (2013) 120:1074–9. 10.1016/j.ophtha.2012.10.022 23380471

[44] French AN Ashby RS Morgan IG Rose KA. Time outdoors and the prevention of myopia. Exp Eye Res. (2013) 114:58–68. 10.1016/j.exer.2013.04.018 23644222

[45] Viallefont V Raftery AE Richardson S. Variable selection and Bayesian model averaging in case-control studies. Stat Med. (2001) 20:3215–30. 10.1002/sim.976 11746314

[46] Łukaszyk E Bień-Barkowska K Bień B. Identification of mortality risks in the advancement of old age: application of proportional hazard models based on the stepwise variable selection and the bayesian model averaging approach. Nutrients. (2021) 13:1098. 10.3390/nu13041098 33801694 PMC8066062

[47] Wang D Zhang W Bakhai A. Comparison of Bayesian model averaging and stepwise methods for model selection in logistic regression. Stat Med. (2004) 23:3451–67. 10.1002/sim.1930 15505893

